# Supplementary figures and images for: Intestinal SIRT1 Deficiency-Related Intestinal Inflammation and Dysbiosis Aggravate TNFα-Mediated Renal Dysfunction in Cirrhotic Ascitic Mice
Source: Int J Mol Sci. 2021 Jan 27;22(3):1233. doi: 10.3390/ijms22031233 (PMC7865325; doi:10.3390/ijms22031233)

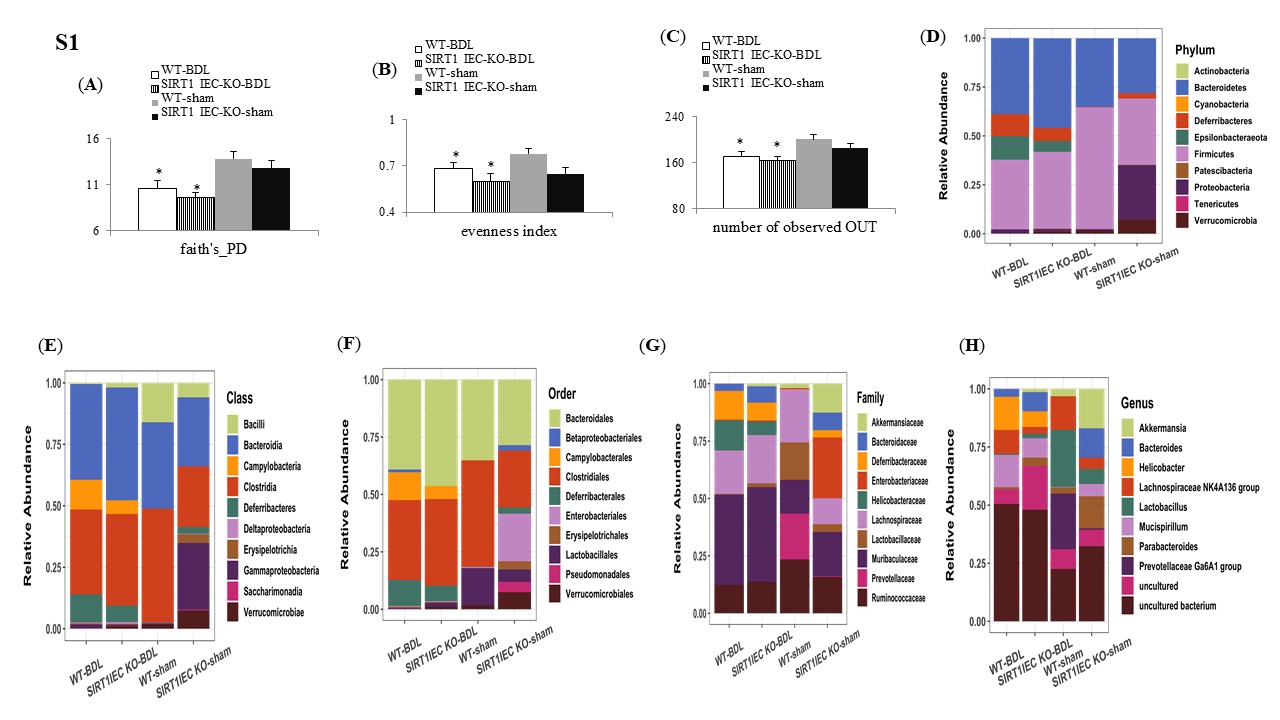

Supplement: Supplementary file 1 [file ijms-22-01233-s001.zip › S 1.JPG]

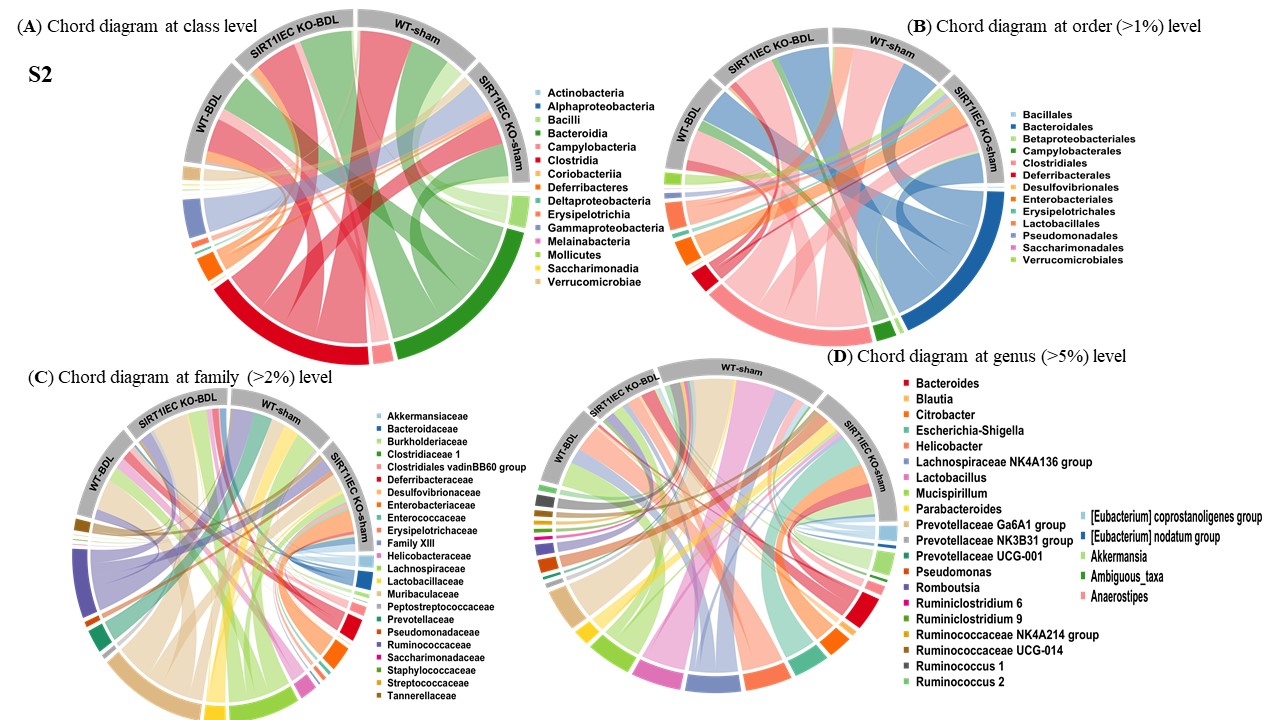

Supplement: Supplementary file 1 [file ijms-22-01233-s001.zip › S 2.JPG]

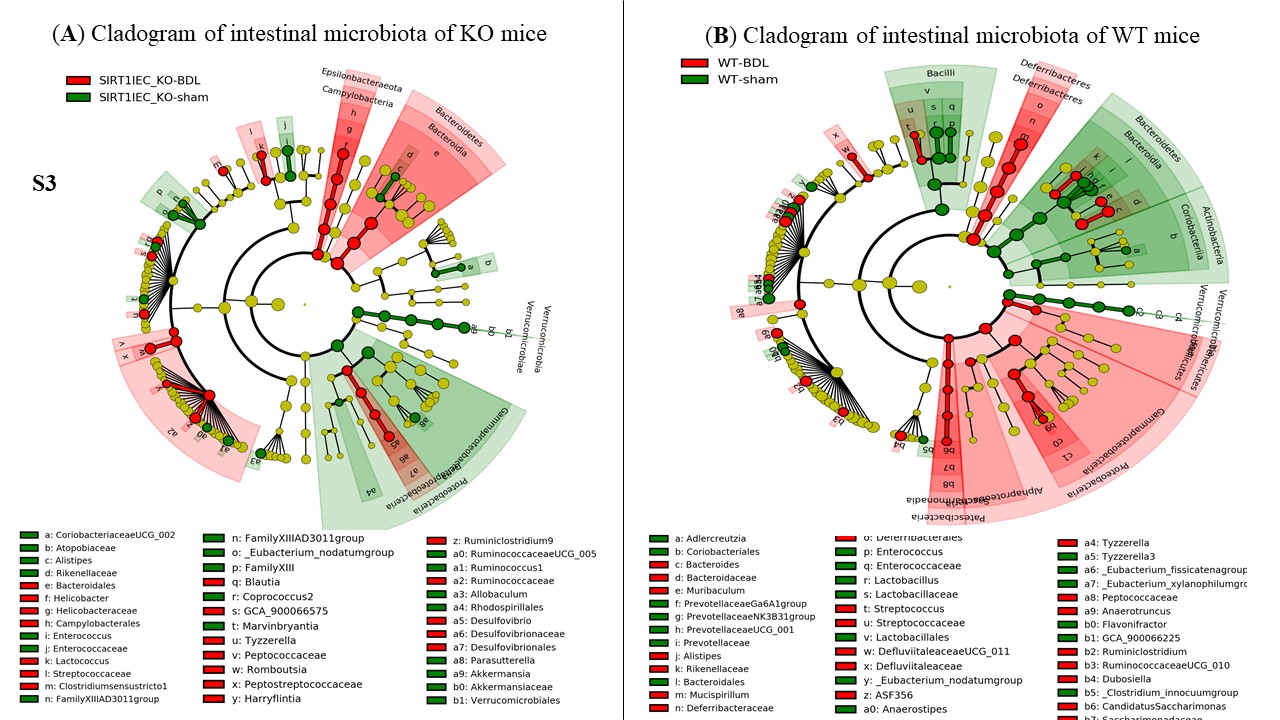

Supplement: Supplementary file 1 [file ijms-22-01233-s001.zip › S 3.JPG]

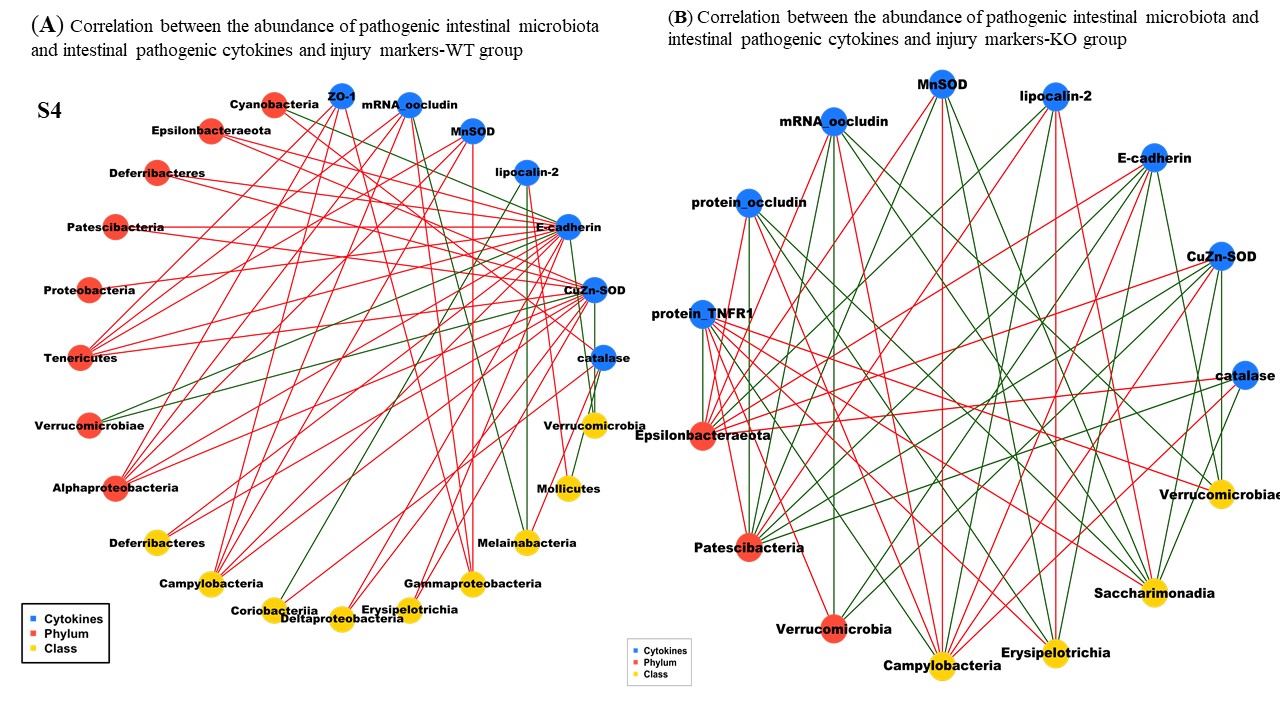

Supplement: Supplementary file 1 [file ijms-22-01233-s001.zip › S 4.JPG]

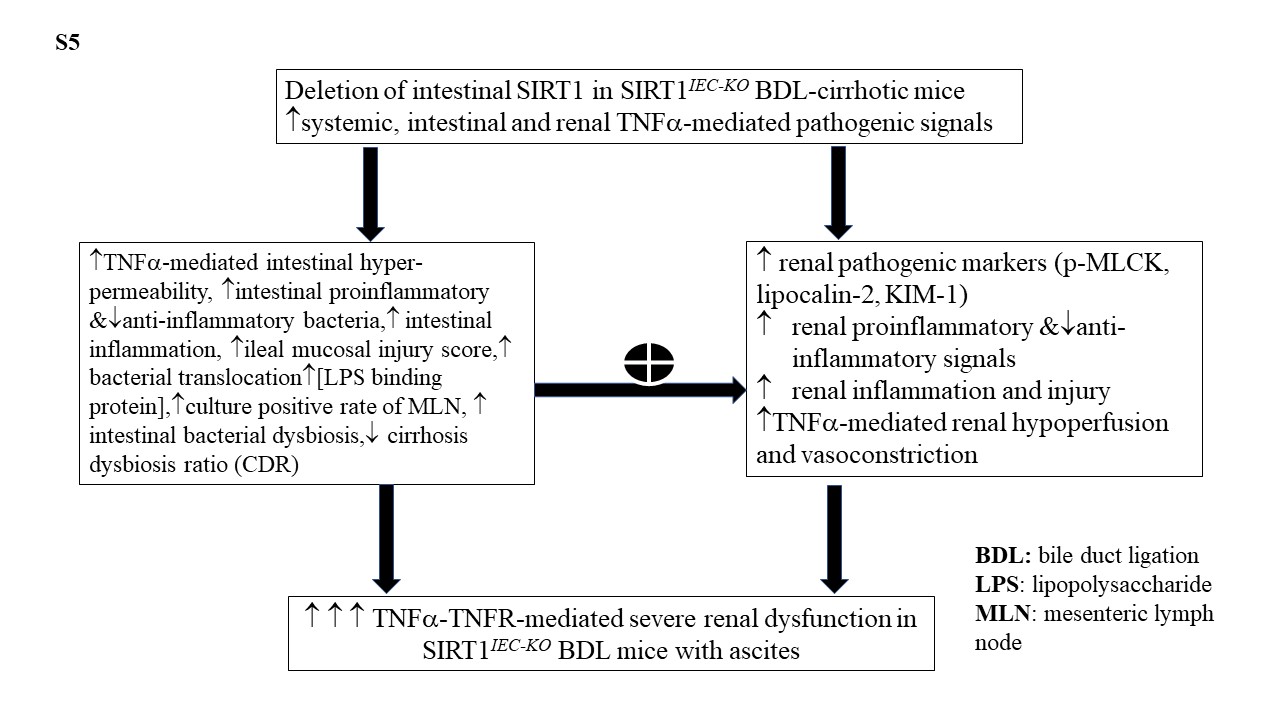

Supplement: Supplementary file 1 [file ijms-22-01233-s001.zip › S 5.JPG]
